# Hazard Curves for Tumor Recurrence and Tumor-Related Death Following Esophagectomy for Esophageal Cancer

**DOI:** 10.3390/cancers12082066

**Published:** 2020-07-27

**Authors:** Joerg Lindenmann, Melanie Fediuk, Nicole Fink-Neuboeck, Christian Porubsky, Martin Pichler, Luka Brcic, Udo Anegg, Marija Balic, Nadia Dandachi, Alfred Maier, Maria Smolle, Josef Smolle, Freyja Maria Smolle-Juettner

**Affiliations:** 1 Division of Thoracic and Hyperbaric Surgery, Department of Surgery, Medical University of Graz, 8036 Graz, Austria; melanie.fediuk@medunigraz.at (M.F.); nicole.neuboeck@medunigraz.at (N.F.-N.); christian.porubsky@medunigraz.at (C.P.); udo.anegg@medunigraz.at (U.A.); alf.maier@medunigraz.at (A.M.); freyja.smolle@medunigraz.at (F.M.S.-J.); 2 Division of Cancer Medicine, Department of Experimental Therapeutics, The University of Texas MD Anderson Cancer Center, UTHealth, Texas A&M College of Medicine, Houston, TX 77030, USA; martin.pichler@medunigraz.at; 3 Division of Oncology, Department of Internal Medicine, Medical University of Graz, 8036 Graz, Austria; marija.balic@medunigraz.at (M.B.); nadia.dandachi@medunigraz.at (N.D.); 4 Diagnostic and Research Institute of Pathology, Medical University of Graz, 8036 Graz, Austria; luka.brcic@medunigraz.at; 5 Department of Orthopaedics and Trauma, Medical University of Graz, 8036 Graz, Austria; maria.smolle@medunigraz.at; 6 Institute of Medical Informatics, Statistics and Documentation, Medical University of Graz, 8036 Graz, Austria; josef.smolle@medunigraz.at

**Keywords:** esophageal cancer, esophagectomy, tumor recurrence, hazard, surveillance, survival

## Abstract

Background: The knowledge of both patterns and risk of relapse following resection for esophageal cancer is crucial for establishing appropriate surveillance schedules. The aim of this study was to evaluate the pattern of hazards for tumor recurrence and tumor-related death in the postoperative long-term follow-up after esophagectomy. Methods: Retrospective single-center analysis of 362 patients, with resected esophageal cancer. Multivariate Cox proportional hazard model was used. Results: A total of 192 (53%) had postoperative tumor recurrence. The relapse patterns of adenocarcinoma and squamous-cell carcinoma showed that each had a single peak, 12 months after surgery. After induction there was one peak at 5 months, the non-induced patients peaked 11 months, postoperatively. At 18 months, the recurrence hazard declined sharply in all cases. The hazard curves for tumor-related death were bimodal for adenocarcinoma, with two peaks at 6 and 22 months and one single peak for squamous-cell carcinoma at 18 months after surgery, showing pronounced decline later on. Conclusion: In curatively resected esophageal cancer, both tumor recurrence hazard and hazard for tumor-related death showed distinct, partly bimodal patterns. It could be justified to intensify the surveillance during the first two postoperative years by initiating a close-meshed follow-up to detect and treat tumor recurrence, as early as possible.

## 1. Introduction

The incidence of esophageal cancer (EC) is still increasing worldwide. Esophageal squamous-cell carcinoma (ESCC) comprises 60–70% of all cases of esophageal cancer. While esophageal adenocarcinoma (EAC) accounts for a further 20–30%, the latter type being more common in developed countries [1]. Basically the conventional treatment modality for the cure of EC is still esophageal resection, either with or without (neo-) adjuvant therapy, depending on the individual tumor stage [2].

However, the prognosis of surgically-treated EC has slightly improved over the past 20 years, though only a minority of tumors qualify for upfront resection.

These selected cases are either unfit for preoperative chemo-radiation, so-called induction therapy, or they present as very early tumor stages. In contrast, ECs with increasing depth of tumor infiltration showed a high incidence of—frequently not clinically detectable—lymph node metastasis. Preoperative treatment was indicated in these cases, because lymph node involvement is still a central prognostic factor, even for postoperative tumor recurrence [3]. In this context, induction therapy corresponding to neo-adjuvant chemo-radiation has emerged as the preferred treatment for locally advanced, resectable EC. In this setting pre-operative chemotherapy is administered intravenously, concurrent to pre-operative external radiation. Induction therapy is associated with improved overall survival (OS) and recurrence free survival (RFS), compared with preoperative chemotherapy or surgery alone [4,5,6,7,8,9,10,11,12].

Despite continuous progress in the multi-modal treatment of resectable EC, the high incidence of postoperative tumor recurrence still remains a serious problem in patients, following curatively resected EC. In this context, it became apparent that more than 80% of tumor recurrences occur within the first 2–3 years after curative esophagectomy [13,14]; the remaining 20% might occur later, until approximately 4 years after surgery [14]. For this reason, conduction of consequent surveillance in the first 5 years after esophagectomy is endorsed by the current guidelines [15,16].

In the meantime, there is growing evidence that 5-year survival following resection of esophageal cancer does not equal “cure”. Even 5-year survivors of EC seem to have a decreasing but persistent residual risk of postoperative tumor recurrence and death from tumor recurrence up to 10 years from diagnosis [17].

In consideration of the low data availability, only few studies dealt with the long-term follow-up after resected EC up till now. These reports usually evaluated staging, the treatment modalities, and the different pattern of postoperative tumor recurrence, respectively [13,14,17,18,19].

In this context, reliable risk estimates of tumor recurrence are of pivotal importance to establish optimal postoperative surveillance strategies, as proposed in case of resected colon cancer, breast cancer, and lung cancer, respectively [20,21,22]. It was shown that the implementation of risk estimates of tumor recurrence is important for the detection of treatable recurrence. In this context, the accurate knowledge of both patterns and relative risk of relapse, forms the basis of an optimal postoperative surveillance schedule.

To our best knowledge, the present study was the first report that dealt with the risk analysis of postoperative tumor recurrence in patients with resected EC, during long-term follow-up. The aim of this retrospective study was to evaluate both patterns of hazards for tumor recurrence and tumor-related death, in the postoperative long-term follow-up of patients with resected EC.

## 2. Results

Among the 362 patients, 86.5% were male, mean aged 62 years. The most frequent tumor localization affected the lower esophageal third in 68.7% of all cases, EAC was the predominating histological subtype in 61.9%. The most common tumor differentiation was G3 (49.8%), and the most common tumor was stage III (30.9%), followed by stage I (24.3%). The most frequent tumor infiltration corresponding to pT3 was confirmed in 44.5% of all cases, pN0 in 51.1%, and R0 was obtained in 87% of all patients. A total of 119 patients (32.9%) underwent induction chemo-radiotherapy and only 61 (17.3%) had adjuvant therapy. Among this small subgroup, 28 (7.7%) received adjuvant chemotherapy, 21 (5.98%) received chemotherapy combined with radiotherapy, and 12 (3.3%) received radiotherapy alone.

Postoperative complications were recorded. According to the Clavien classification scoring from 0 to 5, 26.7%, 0.8%, 8.6%, 32.5%, 25.0%, and 6.4% were recorded among our cohort of patients. The most common complications were mucostasis (32.3%), cardio-respiratory problems (27.9%), and psychosyndrome (21.0%). Anastomotic insufficiency was found in 17.4%.

However, none of these complications had a significant impact on the RFS (Cox proportional hazard model: *p* > 0.05).

A detailed summary of the patients clinico-pathological characteristics, overall and stratified by histology is given in Table 1. According to univariate analysis, statistical significance could be obtained for age, gender, BMI, tumor location, tumor differentiation, tumor infiltration, adjuvant treatment, tumor recurrence, and loco-regional relapse in particular (Table 1).

### 2.1. Survival Data

**Figure 1:**
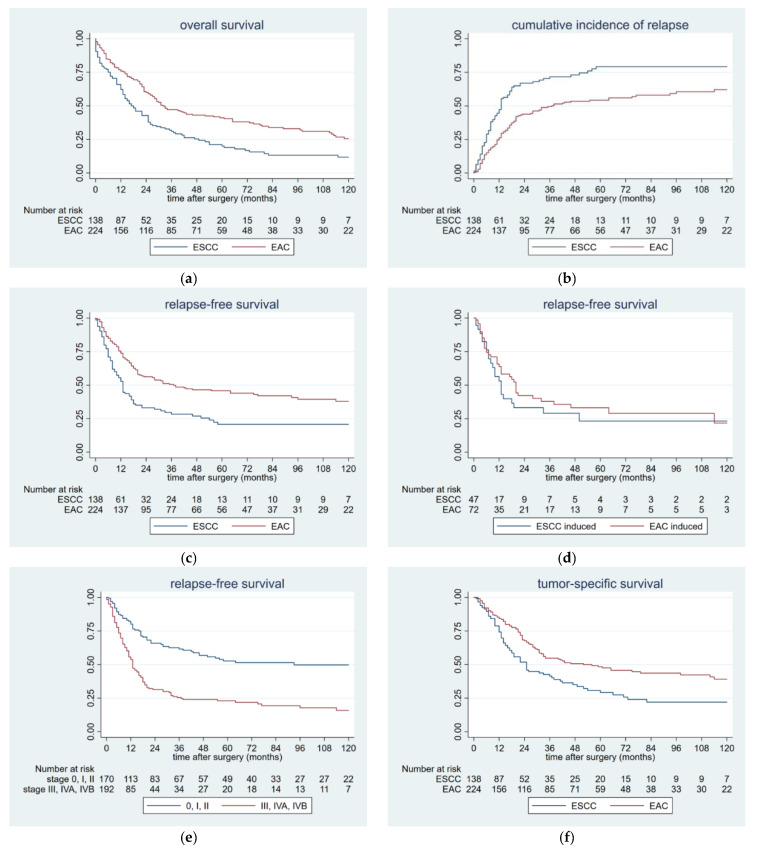
(**a**) Kaplan-Meier-curves showing the relationship between OS and histology in 362 patients with resected esophageal cancer (*p* < 0.0001, Cox proportional hazard model, univariate analysis). (**b**) Times of postoperative tumor recurrence in 362 patients with resected esophageal cancer stratified by histology (*p* < 0.0001, Cox proportional hazard model, univariate analysis). (**c**) Kaplan-Meier-curves comparing RFS and histology in 362 patients with resected esophageal cancer (*p* < 0.0001, Cox proportional hazard model, univariate analysis). **d**) Kaplan-Meier-curves comparing RFS and histology in 119 patients with resected esophageal cancer and induction therapy (*p* = 0.393, Cox proportional hazard model, univariate analysis). (**e**) Kaplan-Meier-curves comparing RFS and tumor stage in 362 patients with resected esophageal cancer (*p* < 0.0001, Cox proportional hazard model, univariate analysis). (**f**) Kaplan-Meier-curves showing the relationship between TSS and histology in 362 patients with resected esophageal cancer (*p* < 0.0001, Cox proportional hazard model, univariate analysis). Abbreviations: OS: Overall survival, EAC: esophageal adenocarcinoma, ESCC: esophageal squamous-cell carcinoma, RFS: Relapse free survival, TSS: Tumor specific survival.

The median follow-up was 22 months (ranging from 0 to 192 months). The 5-year-OS rate was 33.3%. A significant difference was found between those patients with EAC and those with ESCC (41.2% vs. 20.1 %, *p* < 0.0001; Figure 1a). Median survival for the whole cohort was 26 months, compared to 32 months in the EAC group and 17 months in the ESCC group, respectively.

### 2.2. Patterns of Postoperative Tumor Recurrence and Tumor-Specific Survival

Tumor recurrence was found in 192 patients (53%), 160 patients (44.2%) had distant metastasis, 32 (8.8%) had loco-regional relapse. In those patients with distant relapse, the majority (*n* = 95) had EAC and 65 patients had ESCC (*p* = 0.438). Loco-regional recurrence was diagnosed in 20 patients with ESCC, compared to only 12 patients with EAC (*p* = 0.002). (Table 1). The median RFS was 20 months for the whole cohort. In the EAC group, median RFS was 37 months; in the ESCC group, the median was 13 months. One year after surgery, 47% in the ESCC group and 27% in the EAC group developed tumor recurrence. At two years, there were 67% and 43%, and at three years there were 72% and 48%, respectively (*p* < 0.001; Figure 1b).

The 5-year-RFS rate was 37.6% for the whole cohort. Patients with EAC had 46.1%, whereas those with ESCC had 22.0% (*p* < 0.0001; Figure 1c).

A similar pattern could be obtained after stratifying those patients with induction therapy into the histological subgroups. In patients with EAC, the 5-year-RFS was 29.6% compared to 26.1% with ESCC (*p* = 0.393; Figure 1d).

After stratification into the tumor stage, those patients with an early tumor stage had a significantly better prognosis, compared to those with an advanced tumor stage. For those patients with tumor stage 0, I, and II, the 5-year-RFS was 51.8%. Those patients with tumor stage III and IV had 22.6% (*p* < 0.0001; Figure 1e).

From the total sample of patients, 246 died (68%). Out of those 246 patients, the vast majority died from tumor recurrence (*n* = 175; 48.4%) and 71 died from other causes. The median TSS was 36 months, 52 months for those patients with EAC and 25 months for those with ESCC. The 5-year TSS rate was 42.3% for the entire cohort, 48.8% for the EAC group, and 30.7% for the ESCC group, respectively (*p* < 0.0001; Figure 1f).

### 2.3. Risk Factors for Postoperative Tumor Recurrence

Univariate and multivariate analysis of risk factors for the development of postoperative tumor recurrence are displayed in detail in Table 2. The different parameters are presented with respect to the RFS. After application of multivariate analysis, tumor histology, advanced tumor stage, poor tumor differentiation, and no tumor-free resection margin, each showed statistical significance (Table 2).

### 2.4. Hazard Rates for Postoperative Tumor Recurrence

**Figure 2:**
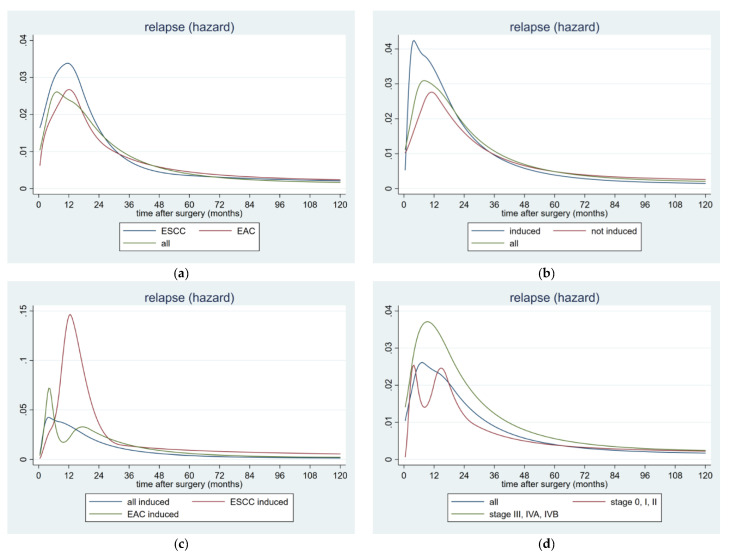
Hazard rate for postoperative tumor recurrence over time in 362 patients with resected esophageal cancer stratified by (**a**) histology and (**b**) induction treatment. (**c**) Hazard rate for postoperative tumor recurrence over time in 119 patients with resected esophageal cancer and induction therapy stratified by histology. (**d**) Hazard rate for postoperative tumor recurrence over time in 362 patients with resected esophageal cancer stratified by early and advanced tumor stage. Abbreviations: EAC: esophageal adenocarcinoma and ESCC: esophageal squamous-cell carcinoma.

When constructing the hazard rate (HR) estimates for the postoperative tumor recurrence, different peaks could be detected for the overall cohort and for both histological subtypes, respectively. Plotting HR over time revealed that the risk of tumor recurrence for the overall sample showed one peak, 7 months after surgery. When stratified into the two histological subtypes, both the relapse pattern of the EAC and the ESCC showed their individual single peak at 12 months, postoperatively. In the ESCC group, the risk of recurrence increased steeply towards a peak (HR > 0.03), one year after resection, after which it decreased to less than half the peak at 2 years. After 18 months, the recurrence hazard declined sharply in all cases (Figure 2a).

After stratification into patients with induction therapy and those without preoperative treatment, a similar pattern could be detected. A very early and prominent peak 5 months after surgery could be confirmed in the induction group, whereas the non-induction group peaked lower and considerably later at 11 months, postoperatively. In the induction group, the risk of recurrence increased steeply towards a peak (HR > 0.04) 5 months after resection, after which it decreased to less than half the peak at 2 years. The peak of the overall sample was between those two, 7 months after surgery. Again in these stratified groups, the recurrence hazard declined sharply after 18 months (Figure 2b).

A similar pattern could be observed in those 119 patients undergoing induction therapy before surgery. After stratification into the histological subtypes, the following pattern could be detected. In the EAC subgroup, the risk of recurrence increased steeply towards a peak (HR > 0.06), 5 months after resection, after which it decreased to less than half the peak at one year. In the ESCC subgroup, the risk of tumor recurrence was considerably higher and therefore increased steeply towards a peak (HR ~0.15), followed by an immediate decrease to less than a fifth of the peak at 30 months (Figure 2c).

After stratification into the tumor stages, the following difference could be noticed. Those patients with advanced tumor stage corresponding stage III and IV had a considerably higher risk for postoperative tumor recurrence, compared with the early tumor stages. In the advanced subgroup, the risk for recurrence showed a striking peak (HR nearly 0.04) at one year after surgery, after which it decreased to less than half the peak at 30 months. In the early group, corresponding tumor stage 0, I, and II, the risk for postoperative tumor recurrence was considerably lower (HR ~0.025) and had a bimodal pattern with two peaks—the first before 6 months and the second before 18 months after surgery (Figure 2d).

### 2.5. Hazard Rates for Postoperative Tumor-Related Death

**Figure 3:**
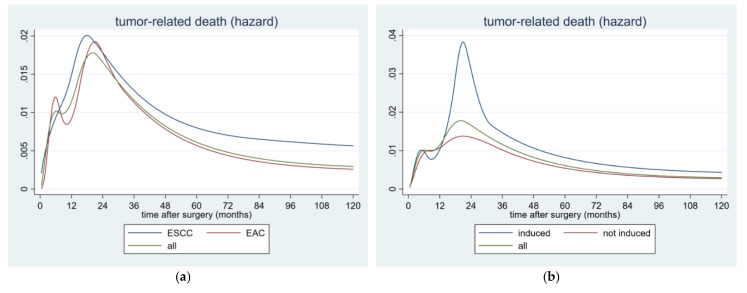
Hazard rate for postoperative tumor-related death in 362 patients with resected esophageal cancer stratified by (**a**) histology and (**b**) induction treatment. Abbreviations: EAC: esophageal adenocarcinoma and ESCC: esophageal squamous-cell carcinoma.

After construction of the HR plots for the postoperative tumor-related death, different patterns could be detected with respect to the histological subtype. The pattern of the EAC was bimodal with one peak at 6 and the second at 22 months, whereas the ESCC had one single, large peak at about 18 months after surgery. The HR for overall tumor-related death also revealed a bimodal pattern, showing the first peak at 6 and the second at 20 months, postoperatively (Figure 3a).

After division into patients with induction therapy and those without preoperative treatment, the bimodal patterns were obtained in both groups. Those patients undergoing pre-operative treatment peaked twice, at 5 and 21 months, compared with those without induction therapy peaking at 7 and 20 months, respectively. Furthermore, the second peak in the induction group was higher than that in the non-induction group, indicating that the induction therapy had no protective effect on the occurrence of a relapse. The overall HR for all these patients reached the first peak at 6 months, followed by the second at 20 months (Figure 3b). However a sharp decline of the tumor-related death could be confirmed in all those cases, after about 30 months after surgery.

## 3. Discussion

This retrospective clinical study showed that in EC, both, the hazard rate for tumor recurrence and the hazard rate for tumor-related death showed distinct, in part bimodal patterns, after esophagectomy. Moreover, 18 months after surgery, the hazard for recurrence diminished rapidly, equaling a sharp flattening of the tumor-related death curve, after 30 months.

Despite continuous progress in the multi-modal treatment of resectable EC, the high incidence of postoperative tumor recurrence still remained a serious problem in patients, following resected EC. In this context, the time and patterns of tumor recurrence after resection for EC were evaluated in several studies. Most reports investigated the incidence of tumor recurrence in patients with resected ESCC [23,24,25,26,27]. Only a few studies analyzed the different recurrence patterns for both histological subtypes, ESCC and EAC, respectively [13,14,17,18,19].

It was shown that in more than 80% of the cases, tumor recurrence occurred within the first postoperative 2 years [13,23] and 3 years, respectively [14,19]. Hiyoshi et al. demonstrated that 88% of those patients with tumor recurrence were diagnosed within the first two years, regardless of the underlying histological subtype [13]. Ninomyia et al. showed that 91.8% of those patients who relapsed after resected ESCC, were detected within two years after surgery [23]. Steffen et al. could confirm similar results for patients with resected EAC, showing a significantly higher incidence of early relapse within two years after surgery, compared to the ESCC subgroup [14].

However these results were consistent with the recurrence patterns of the present study. Among our cohort of 362 patients, the vast majority of the tumor recurrences occurred within the first two postoperative years. In particular, during the first year, both histological subtypes showed a steep increase, followed by a second similar rise up to the end of the second year. Only at the beginning of the third year, the increment changed to a moderate trend, followed by a plateau at the end of the 5th postoperative year. Our results demonstrated impressively that those patients with ESCC had a significantly higher incidence of postoperative tumor recurrence, as compared to those with EAC. In addition, the ESCC group showed both an earlier and steeper rising of relapse, in particular during the first postoperative year (Figure 1b).

As a consequence, those patients with ESCC had a significantly shorter RFS of 13 months, as compared to the EAC subgroup with 37 months. This finding could be mirrored in the significantly different 5-year RFS of 46.1% for EAC and 22.0% for ESCC patients.

In the present study, we demonstrated that patients with EAC had a significantly higher incidence of distant recurrences, whereas patients with ESCC showed a higher incidence of loco-regional recurrence (*p* = 0.002). These findings could be confirmed by other studies [17,18].

Similar comparable results could be obtained with regard to the identified risk factors for postoperative tumor recurrence. Consistent with previous reports, lymph node involvement and increased tumor infiltration corresponding to advanced tumor stage served as independent predictors of postoperative tumor recurrence in the current study [13,18,26,28]. Likewise advanced tumor stage, tumor histology, poor tumor differentiation grade, and no tumor-free resection margin were considered to be statistically significant factors for worse RFS, in our cohort of patients. These findings were favorably in line with other studies [17,19,23,29]. In particular, in case of tumor stage, we could demonstrate that those patients with earlier tumor stages corresponding to UICC 0-II, had a statistically significant better RFS, and tended to recur later, compared to those patients with advanced tumor stages. This finding could be confirmed by the corresponding risk analysis. We were able to show that those patients with advanced tumor stages had a considerably higher risk for tumor recurrence, showing the maximum at the first year after surgery. In contrast, those patients with early tumor stages had a considerably lower risk for relapse.

In addition, our data indicated that OS was significantly influenced by the time to tumor recurrence. In multivariate analysis, a significant association between those risk factors mentioned above and OS, could be proven, confirming the findings as previously reported by Barbetta et al. [17].

In this context, reliable risk analysis of postoperative tumor recurrence seemed to be mandatory for early detection of treatable recurrence, even after esophagectomy for EC. Estimation of HR for postoperative tumor recurrence was already reported for resected colon cancer, breast cancer, and lung cancer [20,21,22], but not for EC, up till now. To our best knowledge the present analysis was the first study dealing with the risk analysis of postoperative tumor recurrence in case of resected EC.

In the current study, we showed that the risk for tumor recurrence increased steeply, until approximately one year after surgery, corresponding to increasing HR. This pattern of early relapse conflicted with the documented time period of 2 postoperative years, as reported by other authors [13,14,19,23]. However the risk of tumor recurrence decreased gradually, thereafter. Two years after surgery, the HR had fallen to half its maximum level.

An interesting aspect could be noticed after stratification into patients with and without induction therapy. Those patients with neoadjuvant chemo-radiation had both, a considerably higher and earlier risk for the development of postoperative tumor recurrence compared to those patients without induction therapy. After splitting the sample of patients following induction therapy in the histological subtypes, those with ESCC had a considerably higher risk for tumor recurrence compared to EAC. Moreover, the induced ESCC group peaked in tumor recurrence at one year after surgery and therefore the peak clearly occurred later than the EAC group.

Similar findings were reported by Robb and team who observed a higher loco-regional recurrence rate, at 5 years, in patients with neoadjuvant chemo-radiotherapy (74%) compared to those with surgery alone (64%) [18].

Steffen et al. reported that in their cohort of patients operated for EC after induction therapy, 71% relapsed within the first two years after surgery [14].

However, there might be three possible explanations for these surprising findings in our cohort of patients, following induction therapy. First, among those patients with neoadjuvant chemo-radiation, advanced tumor stage with increasing T-stage or loco-regional lymph node involvement was present. Second, in those very locally advanced cases that were almost oncological borderline for curative resection, achievement of tumor-free resection margins was not always possible. As a consequence, R1/R2 situations were not preventable. Third, the postoperatively determined tumor regression grading showed lower response rates than preoperatively expected. It might be speculative to find the main reason for this surprising phenomenon but it underlined our assumption that in particular those patients might benefit from closer postoperative surveillance, as proposed by the current study.

Similar findings could be obtained with regard to the HR of the postoperative tumor-related death. In our cohort of patients, the HR of the tumor-related death showed bimodal patterns, irrespective of the analyzed underlying subgroup. The reason for the occurrence of these two peaks remained obscure. The immune-suppressed condition, on the one hand, was enhanced by the surgical trauma, and possible microscopic tumor cell seeding, on the other hand, might serve as potential mechanisms for this second interesting phenomenon. However, further investigations are warranted to clarify these complex interactions between immune-oncology and patterns of tumor recurrence.

With regard to these findings, the knowledge of both patterns and relative risk of relapse might form the basis of an optimal postoperative surveillance schedule tailored to the individual needs of the patient. In this connection, the data of the current study might suggest that we have to pay more attention to the very early postoperative period, in particular, to the first and the second year following radical surgery. This time range was found to be a crucial period showing steep increase of tumor recurrence, as we could show through the estimated HR for relapse and tumor-related death.

However, the optimal surveillance program following radical resection for esophageal cancer is not yet established. There is no uniform consent about the appropriate follow-up management, illustrated by the current surveillance guidelines [15,16,30]. With respect to the findings of the current study, we assumed that patients might benefit from more frequent and close-meshed surveillance, consequently every 3 months, within the first two postoperative years, rather than the current approach of every 3 to 6 months [15]. Furthermore, appropriate imaging such as CT-scan of the chest and the abdomen might be conducted additionally every 3 months, within this proven pivotal time period of increased relapse.

The aim should be to detect postoperative tumor recurrence as early as possible and to start appropriate treatment without further time delay. In this context, early and timely initiation of surgical or oncological treatment of tumor relapse is of utmost importance, to inhibit the progress of further tumor growth. Regarding the findings of the current study, we could demonstrate that patients with advanced tumor stage and induction therapy, in particular, are at high risk for early postoperative tumor recurrence. This subgroup of patients might definitively benefit from this close-meshed surveillance program mentioned above. However, prospective clinical studies are needed to prove that early detection of postoperative tumor recurrence might lead to improved survival, as suggested in previous studies [17].

Finally, there are several limitations in the present study that needs to be stressed on. First, the study was retrospective, observational, and conducted at a single institution, which might introduce bias. Second, due to the heterogeneous collective nature of this study, we could not rule out the presence of some residual confounding by factors that were not included in the analysis, due to not being collected during data ascertainment. Thus, larger prospective multi-centric studies are needed in order to approve these preliminary results and to establish appropriate surveillance regimens and effective treatment strategies for tumor recurrence after resected EC.

## 4. Materials and Methods

Between 1/2004 and 12/2019, 362 patients with EC after esophagectomy with curative intent, were included consecutively in this retrospective single center analysis. The present study was approved by the local ethics committee (Nr. 30-367 ex 17/18). The inclusion criteria were histopathologically proven ESCC or EAC undergoing esophagectomy and reconstruction with gastric pull-up and cervical esophagogastrostomy. Exclusion criteria were palliative tumor stage with distant tumor spread. As this was a retrospective non-intervention study, the institutional review board waived the need for written informed consent from the patients.

The patient-specific data were collected prospectively in the database of our university hospital and were retrospectively extracted for statistical evaluation. Those medical records were reviewed for age, sex, body mass index (BMI), and ASA surgical risk classification (ASA-Physical status; American Society of Anaesthesiologists). The detailed postoperative tumor stage and the incidence of postoperative tumor recurrence during long-term follow-up were documented (Table 1).

### 4.1. Surgery

Standard resection comprised total esophagectomy with a sleeve of the gastric fundus, reconstruction by gastric pull-up, two-field lymph-node dissection, and cervical esophagogastrostomy. For ESCC, esophagectomy was performed using the transthoracic approach, according to McKeown [31]. In case of clinically negative T1 or T2 carcinoma affecting the lower third of the esophagus or the esophagogastric junction, without evidence of para-esophageal lymph-node involvement, trans-hiatal esophagectomy according to Orringer was conducted [32]. The minimally invasive approach (minimally invasive esophagectomy, MIE) applying both, thoracoscopy and laparoscopy, the latter with an additional, small utility incision, was done in those selected patients eligible for MIE. However, intrathoracic esophagogastrostomy according to Ivor-Lewis was not used. Extensive two-field lymphadenectomy was performed routinely in every patient, as previously described [33].

### 4.2. Postoperative Follow-Up

Postoperative tumor staging was done according to the current tumor-node-metastasis (TNM) classification, defined by the Union for International Cancer Control (UICC) and by the American Joint Committee on Cancer (AJCC), 8th edition [2]. Based on this staging result, the decision about both, the individual follow-up and adjuvant treatment was made within the interdisciplinary tumor board.

All patients were postoperatively followed up, according to a modified surveillance program, based on the current guidelines of the National Comprehensive Cancer Network (NCCN) [15] and the European Society of Medical Oncology (ESMO) [16], combined with the German evidence-based S3-guidelines [30]. In this context, regular oncological visits were conducted according to a modified scheme. History and detailed physical investigation, monitoring of the standard tumor marker every 3 months, as well as CT scan of the thorax and abdomen every 6 months for the first two years were performed. Further, history and physical examination were done every 6 months in year three to five. Endoscopy was performed every 4 months for the first two years and thereafter on demand. This follow-up regimen was conducted within a time-range of five years after esophagectomy.

When tumor recurrence was detected during follow-up, the histological diagnosis of the suspicious lesion was established on the basis of histological or cytological sampling. Depending on the site of tumor recurrence, flexible endoscopy, endoscopic ultrasound-guided biopsy, or CT-guided biopsy was done to obtain enough tumor tissue. When this was not possible, definitive radiological evidence of the tumor relapse was required by positron emission tomography (PET) CT.

Basically, tumor recurrence was defined as loco-regional (esophageal bed, anastomotic, or regional lymph nodes) or distant organ metastasis (liver, lung, bones, brain), as previously described [18,34].

Overall survival (OS) was defined as the time from the date of surgery to the date of death from any cause. Relapse free survival (RFS) was calculated from the date of surgery to the date of diagnosis of tumor recurrence. Tumor-specific survival (TSS) was determined from the date of surgery to the date of death due to tumor recurrence.

### 4.3. Data Management

The patient-specific data were collected prospectively in the database of our university hospital and retrospectively extracted for statistical evaluation. The required follow-up data were retrieved from the Regional Health Care System database (openMEDOCS). If a patient did not present for follow-up, the respective primary health care provider was contacted for information. If the patient was suspected to have died, we did a data query at the Austrian Central Obituary Column. This procedure ensured a consistent follow-up until June 2018 or until death. The causes of death were recorded. No patient was lost to follow-up.

### 4.4. Statistical Analysis

Statistical analysis was performed using STATA version 15 (StataCorp, College Station, TX, USA). Besides basic statistics (absolute and relative frequency, median, range), chi²-test and rank-sum-test were applied where appropriate. *p* < 0.05 was considered to indicate statistical significance. Survival curves were prepared according to Kaplan and Meier. Statistical comparisons with regard to survival were performed using the Cox proportional hazard model, either in a univariate approach or in a stepwise forward multivariate procedure. The variables that showed a significant effect in univariate analyses were submitted to multivariate analysis, where only those variables that were significant in the multivariate approach remained in the final model, according to the stepwise forward procedure. Hazard curves were generated according to Lambert and Royston [35].

## 5. Conclusions

Based on the findings of this present study, we are able to demonstrate that in resected esophageal cancer, both tumor recurrence hazard and hazard for tumor-related death showed distinct, partly bimodal patterns. After a steep increase of relapse at the first postoperative year, the hazard for tumor recurrence diminished rapidly after 18 months, equaling a flattening of the tumor-specific survival curve after 30 months. In this context, it might be beneficial to intensify the surveillance in the early postoperative period, in particular within the first and second year after surgery, by initiation of a close-meshed follow-up program to detect and treat tumor recurrence as early as possible.

## Figures and Tables

**Table 1:** Characteristics of 362 patients with resected esophageal cancer. Relationship between clinico-pathological parameters and tumor histology stratified into EAC and ESCC. Abbreviations: EAC: esophageal adenocarcinoma, ESCC: esophageal squamous-cell carcinoma, BMI: Body Mass Index, ASA: ASA-Physical status (American Society of Anaesthesiologists), CRP: preoperative C-reactive protein. Chi^2^-test was used for categorical variables, and rank-sum-test for continuous variables.

Characteristic	Overall	EAC	ESCC	*p* Value
**Number** (percent)	362 (100%)	224 (61.9%)	138 (38.1%)	
**Age** (median; range; years)	62 (22–88)	64 (22–88)	61 (31–82)	0.004
**Gender**				0.003
Male	313 (86.5%)	203 (90.6%)	110 (79.7%)	
Female	49 (13.5%)	21 (9.4%)	28 (20.3%)	
**BMI** (median; range; kg/m^2^)	25.3 (14.9–40.9)	25.7 (14.9–40.9)	24.4 (14.8–35.0)	0.0001
**ASA**				0.316
1	16 (4.4%)	10 (4.5%)	6 (4.3%)	
2	147 (40.6%)	100 (44.6%)	47 (34.1%)	
3	169 (46.7%)	96 (42.9%)	73 (52.9%)	
4	30 (8.3%)	18 (8.0%)	12 (8.7%)	
**CRP** (median; range; mg/L)	3.2 (0.5–260.5)	2.9 (0.5–229.0)	3.55 (0.4–260.5)	0.4648
**Tumor location**				0.001
Upper third	30 (8.3%)	0 (0.0%)	30 (21.7%)	
Middle third	83 (22.9%)	13 (5.8%)	70 (50.7%)	
Lower third/cardia	249 (68.7%)	211 (94.2%)	38 (27.6%)	
**Tumor differentiation**				0.034
G1	17 (4.8%)	15 (6.8%)	2 (1.5%)	
G2	162 (45.4%)	93 (41.9%)	69 (51.1%)	
G3	178 (49.8%)	114 (51.3%)	64 (47.4%)	
N/A	5	2	3	
**Tumor infiltration**				0.003
pT0	24 (6.6%)	10 (4.5%)	14 (10.1%)	
pT1	83 (22.9%)	59 (26.3%)	24 (17.4%)	
pT2	79 (21.8%)	53 (23.7%)	26 (18.8%)	
pT3	161 (44.5%)	98 (43.7%)	63 (45.7%)	
pT4	15 (4.2%)	4 (1.8%)	11 (8.0%)	
**Lymph node involvement**				0.143
pN0	185 (51.1%)	108 (48.2%)	77 (55.8%)	
pN1	106 (29.3%)	65 (29.0%)	41 (29.7%)	
pN2	41 (11.3%)	27 (12.1%)	14 (10.1%)	
pN3	30 (8.3%)	24 (10.7%)	6 (4.4%)	
**Tumor stage**				0.001
0	9 (2.5%)	9 (4.0%)	0 (0.0%)	
I	88 (24.3%)	51 (22.8%)	37 (26.8%)	
II	73 (20.2%)	26 (11.6%)	47 (34.0%)	
III	112 (30.9%)	79 (35.3%)	33 (23.9%)	
IVA	60 (16.6%)	49 (21.9%)	11 (8.0%)	
IVB	20 (5.5%)	10 (4.4%)	10 (7.3%)	
**Resection margin**				0.078
R0	293 (86.9%)	186 (89.4%)	107 (82.9%)	
R1	39 (11.6%)	21 (10.1%)	18 (14.0%)	
R2	5 (1.5%)	1 (0.5%)	4 (3.1%)	
N/A	25	16	9	
**Neo-adjuvant treatment**				0.706
Yes	119 (32.9%)	72 (32.1%)	47 (34.1%)	
No	243 (67.1%)	125 (67.9%)	91 (65.9%)	
**Adjuvant treatment**				0.017
Yes	61 (17.3%)	30 (13.6%)	31 (23.5%)	
No	292 (87.7%)	191 (86.4%)	101 (76.5%)	
N/A	9	3	6	
**Tumor recurrence**				0.005
Total number of recurrences	192 (53%)	107 (29.6%)	85 (23.5%)	
Loco-regional	32 (8.8%)	12 (6.3%)	20 (10.4%)	0.002
Distant metastases	160 (44.2%)	95 (49.5%)	65 (33.9%)	0.438

**Table 2:** Cox regression model analysis for risk factors of postoperative tumor recurrence in 362 patients with esophageal cancer undergoing esophagectomy and reconstruction with gastric pull-up and cervical esophagogastrostomy. Abbreviations: HR: hazard ratio, SE: standard error, CI: confidence interval, BMI: Body Mass Index, ASA: ASA-Physical status (American Society of Anaesthesiologists), CRP: preoperative C-reactive protein, RFS: relapse free survival. Cox’ proportional hazards model was used as a statistical test, in both univariate and multivariate (stepwise forward) analysis. The 11 variables which were significant in the univariate analysis were entered together into the multivariate (stepwise forward) analysis, where only four of them remained in the final model.

Characteristic	HR	SE	95% CI	*p* Value
**RFS**				
**Univariate Analysis**				
Age	0.999	0.006	0.986–1.013	0.968
Gender	1.302	0.295	0.834–2.030	0.244
BMI	0.980	0.016	0.947–1.013	0.247
ASA	1.259	0.134	1.002–1.551	0.030
CRP	1.010	0.002	1.004–1.015	<0.001
Albumin	0.689	0.100	0.518–0.916	0.011
Histology	1.874	0.275	1.405–2.499	<0.001
Tumor location	0.594	0.063	0.482–0.733	<0.001
Tumor differentiation	1.705	0.219	1.324–2.194	<0.001
Tumor infiltration	1.675	0.134	1.432–1.960	<0.001
Lymph node involvement	1.763	0.125	1.533–2.027	<0.001
Tumor stage	1.580	0.097	1.400–1.784	<0.001
Resection margin	2.312	0.370	1.688–3.166	<0.001
Neo-adjuvant treatment	1.341	0.207	0.990–1.817	0.057
Adjuvant treatment	1.653	0.284	1.181–2.314	0.003
**Multivariate Analysis**				
Histology	1.956	0.315	1.426–2.683	<0.001
Tumor stage	1.592	0.110	1.389–1.824	<0.001
Tumor differentiation	1.426	0.200	1.082–1.879	0.012
Resection margin	1.635	0.277	1.782–2.280	0.004
